# Health risk assessment of nitrate in groundwater resources of Iranshahr using Monte Carlo simulation and geographic information system (GIS)

**DOI:** 10.1016/j.mex.2019.07.024

**Published:** 2019-07-31

**Authors:** Naseh Shalyari, Abdolazim Alinejad, Amir Hossein Ghazizadeh Hashemi, Majid RadFard, Mansooreh Dehghani

**Affiliations:** a Department of Environmental Health Engineering, School of Public Health, Tehran University of Medical Sciences, Tehran, Iran; b Department of Public Health, Fasa University of Medical Sciences, Fasa, Iran; c Department of Otolaryngology, Loghman Educational Hospital, School of Medicine, Shahid Beheshti, University of Medical Sciences, Tehran, Iran; d Department of Environmental Health Engineering, School of Public Health, Shiraz University of Medical, Sciences, Shiraz, Iran

**Keywords:** Human health, Risk assessment of nitrate, Uncertainty measurement, Monte-Carlo Simulation, GIS, Sistan and Baluchistan

## Abstract

Because of exposure to a wide range of chemical contaminants such as nitrate via potable water resources, the use of the approaches to set standards for drinking water quality and also to do a risk assessment is necessary for maintaining the public health. High levels of nitrate in drinking water can have adverse health effects; primarily for infants and pregnant women. So, the present study aimed to the assessment of nitrate health risk in drinking water resources of the Iranshahr city, Sistan and Baluchistan province and also, evaluation of the uncertainty of nitrate and the probability of contamination occurrence by Monte-Carlo Simulation (MCS) technique. Besides, the geographic information system (Arc GIS, Ver 10.3) was applied to mapping the nitrate concentration in groundwater resources of the studied area. For these aims, the numbers of 66 samples were collected from rural groundwater resources, and nitrate concentration was measured using a Spectrophotometer in wavelength of 220 nm. According to the results, the nitrate concentration was in the range of 6.49 mg/L, and its average level was 6.15 mg/L. Also, the simulation results with 90% confidence showed that the hazard equitant (HQ) in the infant groups, children-teenagers and adults was less than 0.331, 0.311, 0.312, and 0.3, respectively.

## Graphical abstract

**Figure:**
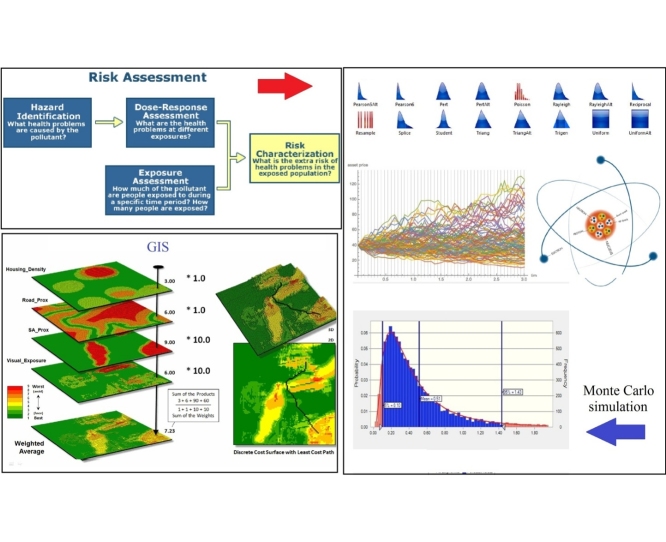

**Specification Table**

**Table:** 

Subject area	Environmental Science
More specific subject area	Nitrate pollution
Protocol name	A nitrate risk assessment by Monte Carlo simulation (MCS)
How data were acquired	Sixty-six samples were collected from rural water resources and analyzed using UV visible Spectrophotometer (DR/5000). All experiments were with two-time repetition.
Reagents/tools	Spectrophotometer (DR/5000, USA), pH meter (model wtw), Simulation by the Monte Carlo Analysis (Crystal Ball ribbon), Spatial distribution Arc GIS (Ver. 10.3)
Data source location	Iranshahr city, Sistan and Baluchistan Province, Iran
Data accessibility	Data is presented in this article.
Trial registration	Not applicable
Ethics	Not applicable

## Value of the protocol

**Table:** 

• The adverse health effects of exposure to nitrate in drinking water resources can be determined by using health risk assessment methods. This protocol introduces a practical and straightforward method for nitrate risk assessment in different exposed groups, including infants, children, teenagers, and adults. • Monte-Carlo simulation (MCS) or probability simulation is one of the most comprehensive approaches of probabilistic modeling that can be used to explain the impact of risk and uncertainty in forecasting models. • In the present research, a probabilistic risk assessment of nitrate exposure was carried out for different exposed groups. This protocol is easy to follow and understand.

## Description of protocol

### Background

In recent decades, with the rapid population growth, increasing human activities especially agriculture and industrial development; contamination of groundwater resources, as the primary source of drinking water, by various chemicals has created a severe concern to human health in almost all regions of the world [1], [2], [3]. Continual exposure to nitrate, as one of the main pollutants in groundwater reservoirs, leading to adverse health effects such as methemoglobinemia (blue baby syndrome) [4], particularly in infants groups [5], [6], [7]. So, monitoring the groundwater resources, and also the use of the approaches to health risk assessment of water contaminants should be necessary for health promotion programs.

The United States Environment Protection Agency (USEPA) defines the human health risk assessment as the systematic approach for an estimating the likelihood of adverse health effects in the exposed population who may be susceptive to specific harmful substances in polluted ecological systems, such as water resources [8]. This approach presents a systematic pattern of the quantitative or semi-quantitative description of environmental health effects caused by exposure to deleterious substances [9], [10]. Several studies have been paid attention to risk assessment models [11], [12]. In risk assessment studies, because of imprecision and insufficiency of the environmental data, two factors should be taken into account: (1) data uncertainty, (2) uncertainty measurement [13]. An uncertainty, which is an inevitable part of risk assessment refers to the situation of limited knowledge about the real value of a parameter or variable [10], [14]. However, it can be quantified, evaluated, and reliably modeled by applying different technical methods [13], [15], [16]. One such method is the Monte-Carlo Simulation (MCS) technique.

MCS (what-if analysis) – as one of the most broadly used methods for probabilistic risk assessment (PRA) modeling – is an approach that can evaluate the variability, heterogeneity, and uncertainty in the several parameters of the human health risk assessment procedure [14], [17]. In this probabilistic approach, all the parameters used in assessing the risk are considered as distributions to achieve a wide range of outcomes (a risk or hazard quotient) after repeated simulations usually 10,000 or more [18], [19]. A simple method to perform MCS is to create the model in Microsoft Excel, then use of Crystal Ball® ribbon [17].

Keeping this in preset, the aims of present protocol were: (1) to evaluate the nitrate concentrations in the groundwater resources of the Iranshahr area, (2) to assess the human health risk for different exposed groups with both deterministic and probabilistic point of view, (3) MCS technique (by using of Crystal Ball® software) was utilized for sensitivity analysis and quantification of the uncertainties related to the risk, (4) the spatial distribution of nitrate concentration in the study area was carried out using the Inverse distance weighted (IDW) method in the ArcGIS (Ver 10.3) software [20].

### Study area

**Fig. 1:**
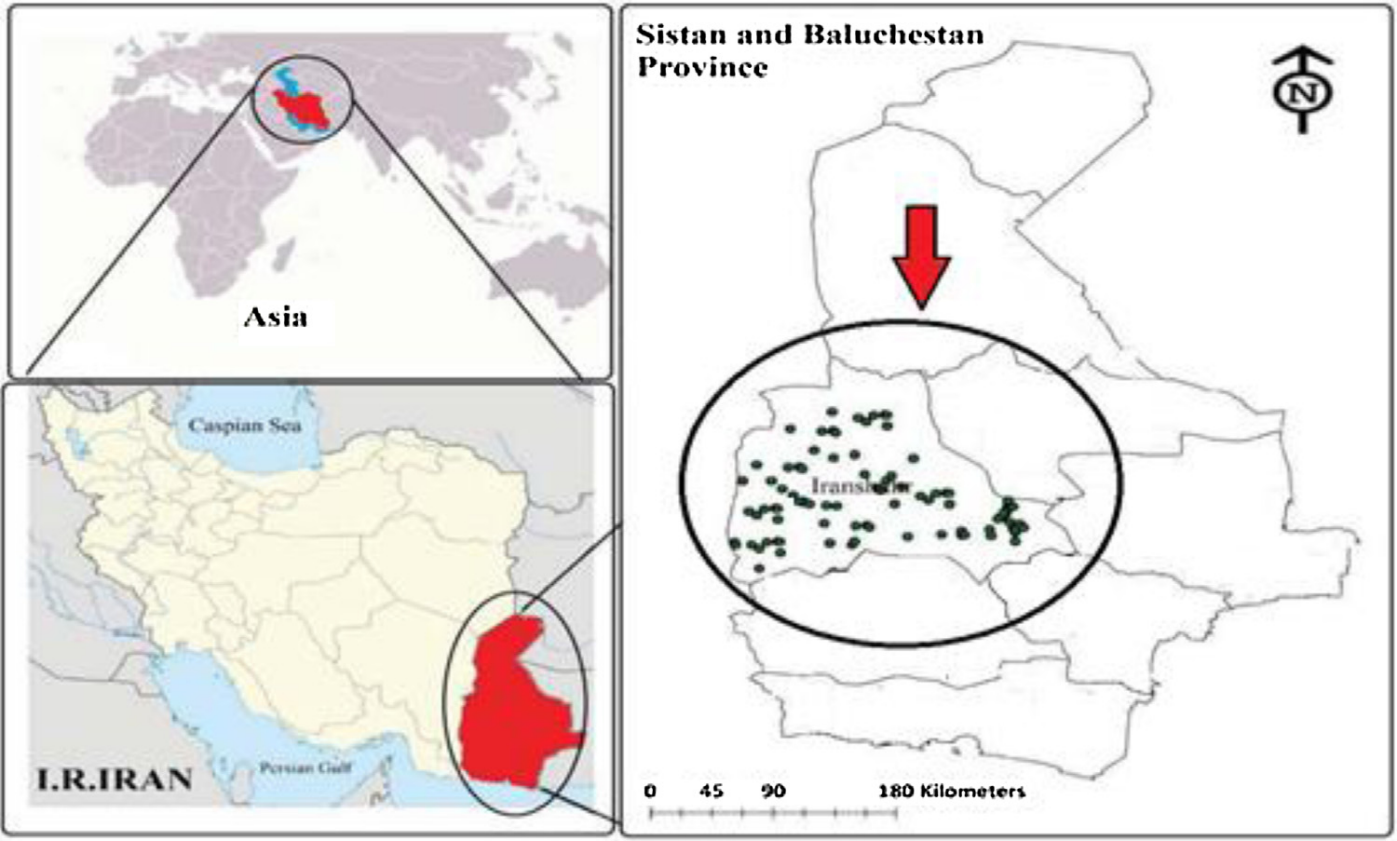
The geographical location of the study area and sampling points.

Fig. 1 shows the geographic location of the studied area and sampling points in rural drinking water resources of Iranshahr city, Sistan, and Baluchistan province.

The study area located in the Sistan and Baluchistan province, southeast of Iran and has the dry and hot climate. The highest and lowest air temperatures are 50 °C and −6.2 °C, respectively, and also, the yearly mean temperature of this region is 32 °C. Iranshahr city located between 27°12′09′ N latitude 60°41′05′ E longitudes, encompassing an area of 30,200 km^2^. This city has a hot desert climate with extremely hot summers and mild winters and it average height above sea level is about 519 m. The total population of the region is 131,232 peoples [21]. The majority occupation of the people of this county is farming. Since nitrogen is a vital nutrient for most plants, nitrate will play a fundamental role in agriculture activities, subsequently, in groundwater resources contamination.

### Sampling procedure

In this cross-sectional study, sixty-six groundwater samples were collected from groundwater resources of rural areas of Iranshahr city, which are being frequently used for drinking water supply (Fig. 1). Before sampling, all dug-wells were pumped for about fifty minutes to remove the influence of stagnant water. All containers of groundwater sample (polyethylene containers with 1 L capacity) were rinsed four times, by deionized water, before being sampled. In the following, the samples were labeled, stored at 4 °C and transported to the laboratory for chemical analysis of essential parameters. All analysis carried out according to Standard Methods for Examination of Water and Wastewater [22].

### Laboratory experiment

According to guidelines, delivery time between sample collection and laboratory receipt was about 6–7 [22]. Nitrate concentration was analyzed using UV Vis Spectrophotometer (HACH DR/5000) in the wavelength of 220 [23], [24]. All specific analysis was carried out according to standard methods for examination water and wastewater [22]. Also, it should be noted that all experiments were carried out twice. The measured concentration of nitrate is shown in Table 2.

### Data analysis

Correlation analysis was done by the Pearson correlation coefficient. All data has been surveyed using statistical package IBM SPSS Version 16.00 (SPSS Inc., Chicago, IL, USA). Also, significance tests were at 95% of confidence level.

### Spatial distribution and interpolation

Interpolation forecasts values for cells in a raster from a restricted number of sample data points. It can be used to forecast unknown values for any spatial point data, such as elevation, rainfall, chemical concentrations, and so on [25]. Several interpolation techniques are often used in the atmospheric sciences such as inverse distance weighted (IDW) and kriging [26].

IDW is deterministic method by considering the surrounding points. The assumption of this method is that interpolation values will be more similar to near sample data than farther ones. The weight will change linearly according to sample data distance [27]. Kriging is similar stochastic approximation to IDW which use linear combination of weight to estimate the value among sample data [28]. The assumption of this method is the distance among sample data showing the important geographical correlation on the interpolation result [29]. IDW provide more accurate interpolation result than the kriging [30].

**Fig. 2:**
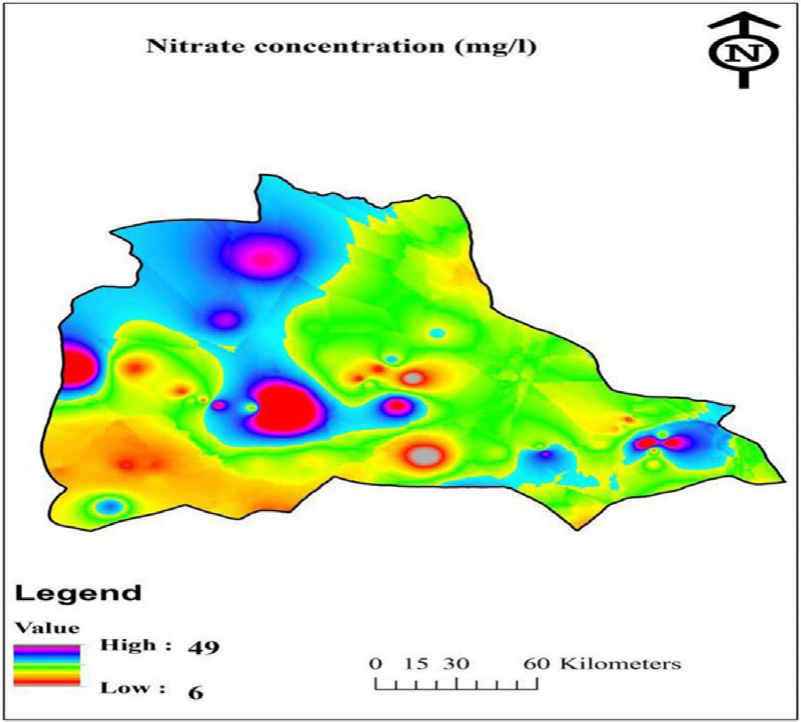
Spatial distribution of nitrate in Iranshahr city.

In present study, GIS software and IDW interpolation method [31] (Fig. 2) were employed for drawing the spatial distribution of nitrate concentration in the study area [32]. Interpolation is the method that uses known data values to estimate unknown data values. IDW is an advanced geostatistical method that can be used for the analysis of spatial correlations, and also it can construct the prediction maps of any unsampled value [32]. Fig. 2 shows the spatial distribution of nitrate in Iranshahr city.

### Risk assessment of nitrate

Nitrate pollution is a significant concern in most groundwater resources in Iran, as well as many parts of the world [8]. The health risk assessment is the systematic framework for estimating the likelihood of adverse health effects in the exposed population who may be susceptive to specific harmful substances in polluted ecological systems [9].

In the present study, a risk assessment was carried out in four groups of the exposed population including an infant (<2 years), children (2–6 years), teenager (6–16 years), and adult (>16 years). First, the daily nitrate consumption was calculated by Eq. (1):

(1) $$\text{EDI}=\frac{C_{f}\times C_{d}}{B_{w}}$$

where EDI: estimation of daily nitrate consumption (mg/kg), *C*_*f*_: nitrate concentration in drinking water (mg/L), *C*_*d*_: average daily drinking water intake, *B*_*w*_: body weight (kg).

Non-carcinogenic impact of a single element can be stated as hazard quotient (HQ) using Eq. (2):

(2) $$\text{HQ}=\frac{\text{EDI}}{\text{RFD}}$$

The RFD is the reference dose of a specific pollutant which is expressed in mg/kg body weight (BW) per day. The reference dose (RFD) is of great significance in the non-carcinogenic risk assessment. According to the database of Integrated Risk Information System guideline, the amount of RFD for NO_3_^−^ is 1.6 mg/kg BW day for nitrate from the digestive tract [8], [33]. The value of HQ < 1 indicates that the harmful effects of exposure cannot be expected, but HQ > 1, indicates that the non-carcinogenic risk excesses the acceptable level [34]. The values of formula parameters for different exposed groups have been shown in Table 1.

**Table 1:** Values of parameters which are used in risk assessment.

Group	*C*_*f*_	*C*_*d*_	*B*_*w*_	RFD	Reference
Infant	–	0.08	10	1.6	[32]
Children	–	0.85	15	1.6	[34]
Teenager	–	2	50	1.6	[35]
Adults	–	2.5	78	1.6	[7]
Unit	mg/L	L/day	kg	mg/kg day	–

The nitrate concentration and also, calculated hazard quotient for drinking water samples are presented in Table 2.

**Table 2:** Calculated hazard quotient for different groups.

No.	Nitrate concentration (mg/L)	EDI				HQ			
		Infants	Children	Teenagers	Adults	Infants	Children	Teenagers	Adults
1	12.000	0.0960	0.6800	0.4800	0.3846	0.0600	0.4250	0.3000	0.2404
2	18.500	0.1480	1.0483	0.7400	0.5929	0.0925	0.6552	0.4625	0.3706
3	24.000	0.1920	1.3600	0.9600	0.7692	0.1200	0.8500	0.6000	0.4808
4	14.000	0.1120	0.7933	0.5600	0.4487	0.0700	0.4958	0.3500	0.2804
5	9.000	0.0720	0.5100	0.3600	0.2885	0.0450	0.3188	0.2250	0.1803
6	12.000	0.0960	0.6800	0.4800	0.3846	0.0600	0.4250	0.3000	0.2404
7	13.500	0.1080	0.7650	0.5400	0.4327	0.0675	0.4781	0.3375	0.2704
8	17.500	0.1400	0.9917	0.7000	0.5609	0.0875	0.6198	0.4375	0.3506
9	15.000	0.1200	0.8500	0.6000	0.4808	0.0750	0.5313	0.3750	0.3005
10	17.000	0.1360	0.9633	0.6800	0.5449	0.0850	0.6021	0.4250	0.3405
11	20.000	0.1600	1.1333	0.8000	0.6410	0.1000	0.7083	0.5000	0.4006
12	28.000	0.2240	1.5867	1.1200	0.8974	0.1400	0.9917	0.7000	0.5609
13	14.500	0.1160	0.8217	0.5800	0.4647	0.0725	0.5135	0.3625	0.2905
14	13.000	0.1040	0.7367	0.5200	0.4167	0.0650	0.4604	0.3250	0.2604
15	17.600	0.1408	0.9973	0.7040	0.5641	0.0880	0.6233	0.4400	0.3526
16	14.500	0.1160	0.8217	0.5800	0.4647	0.0725	0.5135	0.3625	0.2905
17	10.000	0.0800	0.5667	0.4000	0.3205	0.0500	0.3542	0.2500	0.2003
18	6.500	0.0520	0.3683	0.2600	0.2083	0.0325	0.2302	0.1625	0.1302
19	16.500	0.1320	0.9350	0.6600	0.5288	0.0825	0.5844	0.4125	0.3305
20	15.000	0.1200	0.8500	0.6000	0.4808	0.0750	0.5313	0.3750	0.3005
21	8.500	0.0680	0.4817	0.3400	0.2724	0.0425	0.3010	0.2125	0.1703
22	6.500	0.0520	0.3683	0.2600	0.2083	0.0325	0.2302	0.1625	0.1302
23	9.000	0.0720	0.5100	0.3600	0.2885	0.0450	0.3188	0.2250	0.1803
24	18.500	0.1480	1.0483	0.7400	0.5929	0.0925	0.6552	0.4625	0.3706
25	12.000	0.0960	0.6800	0.4800	0.3846	0.0600	0.4250	0.3000	0.2404
26	13.000	0.1040	0.7367	0.5200	0.4167	0.0650	0.4604	0.3250	0.2604
27	16.500	0.1320	0.9350	0.6600	0.5288	0.0825	0.5844	0.4125	0.3305
28	10.000	0.0800	0.5667	0.4000	0.3205	0.0500	0.3542	0.2500	0.2003
29	13.500	0.1080	0.7650	0.5400	0.4327	0.0675	0.4781	0.3375	0.2704
30	25.000	0.2000	1.4167	1.0000	0.8013	0.1250	0.8854	0.6250	0.5008
31	12.000	0.0960	0.6800	0.4800	0.3846	0.0600	0.4250	0.3000	0.2404
32	12.000	0.0960	0.6800	0.4800	0.3846	0.0600	0.4250	0.3000	0.2404
33	13.500	0.1080	0.7650	0.5400	0.4327	0.0675	0.4781	0.3375	0.2704
34	17.000	0.1360	0.9633	0.6800	0.5449	0.0850	0.6021	0.4250	0.3405
35	49.000	0.3920	2.7767	1.9600	1.5705	0.2450	1.7354	1.2250	0.9816
36	17.000	0.1360	0.9633	0.6800	0.5449	0.0850	0.6021	0.4250	0.3405
37	19.500	0.1560	1.1050	0.7800	0.6250	0.0975	0.6906	0.4875	0.3906
38	11.500	0.0920	0.6517	0.4600	0.3686	0.0575	0.4073	0.2875	0.2304
39	15.000	0.1200	0.8500	0.6000	0.4808	0.0750	0.5313	0.3750	0.3005
40	12.500	0.1000	0.7083	0.5000	0.4006	0.0625	0.4427	0.3125	0.2504
41	18.000	0.1440	1.0200	0.7200	0.5769	0.0900	0.6375	0.4500	0.3606
42	14.500	0.1160	0.8217	0.5800	0.4647	0.0725	0.5135	0.3625	0.2905
43	13.000	0.1040	0.7367	0.5200	0.4167	0.0650	0.4604	0.3250	0.2604
44	15.500	0.1240	0.8783	0.6200	0.4968	0.0775	0.5490	0.3875	0.3105
45	11.500	0.0920	0.6517	0.4600	0.3686	0.0575	0.4073	0.2875	0.2304
46	14.500	0.1160	0.8217	0.5800	0.4647	0.0725	0.5135	0.3625	0.2905
47	11.000	0.0880	0.6233	0.4400	0.3526	0.0550	0.3896	0.2750	0.2204
48	13.000	0.1040	0.7367	0.5200	0.4167	0.0650	0.4604	0.3250	0.2604
49	15.800	0.1264	0.8953	0.6320	0.5064	0.0790	0.5596	0.3950	0.3165
50	16.500	0.1320	0.9350	0.6600	0.5288	0.0825	0.5844	0.4125	0.3305
51	6.000	0.0480	0.3400	0.2400	0.1923	0.0300	0.2125	0.1500	0.1202
52	16.000	0.1280	0.9067	0.6400	0.5128	0.0800	0.5667	0.4000	0.3205
53	15.500	0.1240	0.8783	0.6200	0.4968	0.0775	0.5490	0.3875	0.3105
54	16.000	0.1280	0.9067	0.6400	0.5128	0.0800	0.5667	0.4000	0.3205
55	13.000	0.1040	0.7367	0.5200	0.4167	0.0650	0.4604	0.3250	0.2604
56	13.000	0.1040	0.7367	0.5200	0.4167	0.0650	0.4604	0.3250	0.2604
57	10.000	0.0800	0.5667	0.4000	0.3205	0.0500	0.3542	0.2500	0.2003
58	12.500	0.1000	0.7083	0.5000	0.4006	0.0625	0.4427	0.3125	0.2504
59	23.500	0.1880	1.3317	0.9400	0.7532	0.1175	0.8323	0.5875	0.4708
60	13.000	0.1040	0.7367	0.5200	0.4167	0.0650	0.4604	0.3250	0.2604
61	26.500	0.2120	1.5017	1.0600	0.8494	0.1325	0.9385	0.6625	0.5308
62	8.500	0.0680	0.4817	0.3400	0.2724	0.0425	0.3010	0.2125	0.1703
63	20.500	0.1640	1.1617	0.8200	0.6571	0.1025	0.7260	0.5125	0.4107
64	14.000	0.1120	0.7933	0.5600	0.4487	0.0700	0.4958	0.3500	0.2804
65	13.000	0.1040	0.7367	0.5200	0.4167	0.0650	0.4604	0.3250	0.2604
66	12.500	0.1000	0.7083	0.5000	0.4006	0.0625	0.4427	0.3125	0.2504

Mean	6.15	0.0492	0.3484	0.2459	0.1970	**0.0307**	**0.2177**	**0.1537**	**0.1232**
Min	6.00	0.0480	0.3400	0.2400	0.1923	**0.0300**	**0.2125**	**0.1500**	**0.1202**
Max	49.00	0.3920	2.7767	1.9600	1.5705	**0.2450**	***1.7354***	***1.2250***	**0.9816**
SD	6.15	0.0492	0.3484	0.2459	0.1970	**0.0307**	**0.2177**	**0.1537**	**0.1232**

### Monte Carlo simulation (MCS) and Crystal Ball ribbon

MCS (what-if analysis) – as one of the most broadly used methods for probabilistic risk assessment (PRA) modeling – is an approach that can evaluate the variability and uncertainty in the several parameters of the human health risk assessment procedure [14]. In the present study, the variability and sensitivity analysis of the predictions of the risk assessment model was carried out by using the Monte Carlo simulation technique. A simplified approach to perform MCS is to create the model without uncertainty in Microsoft Excel software, then use the spreadsheet-based application, such as Crystal Ball® software [36], [37].

**Fig. 3:**
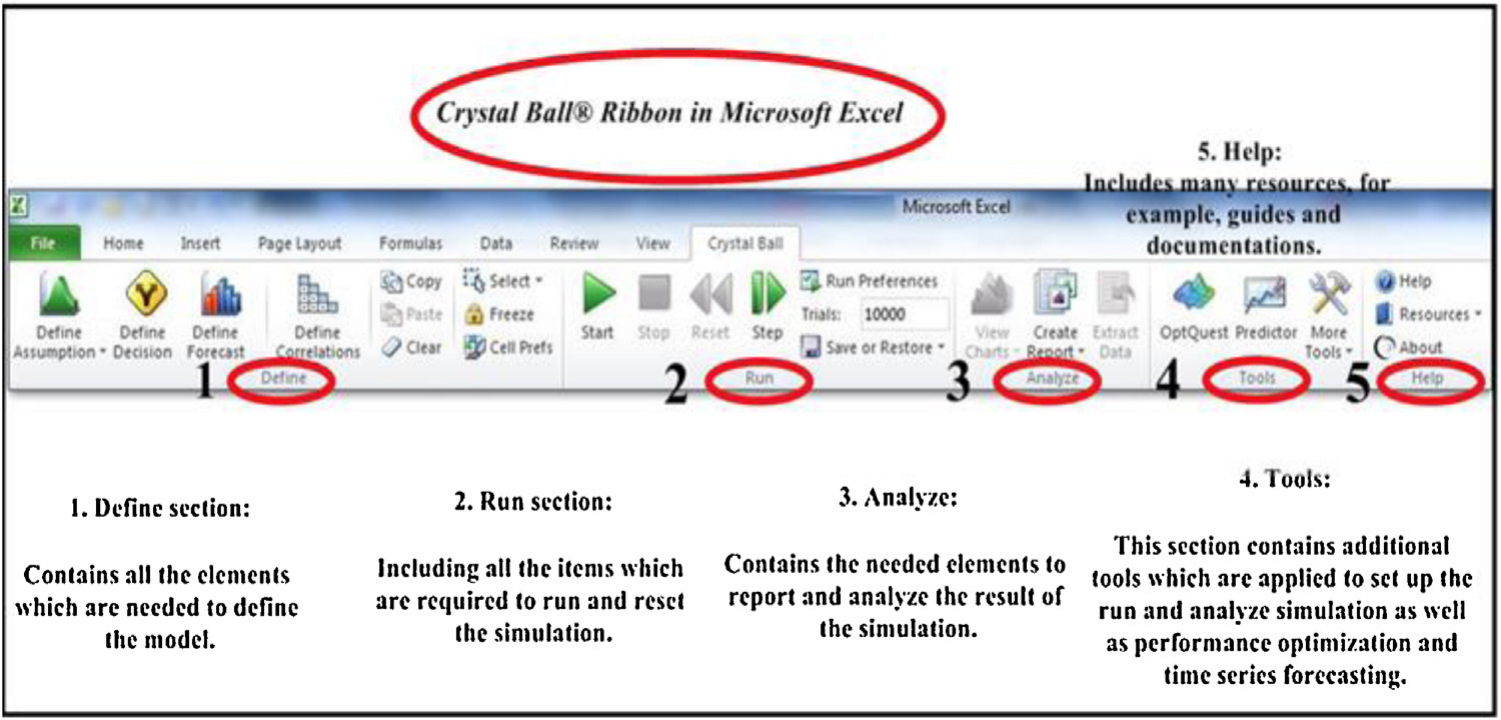
The Status bar of Crystal Ball® ribbon in Microsoft Excel.

Crystal Ball is an “Add-in” for Microsoft Excel that is used to perform analyze, produce the input distribution values, collect the output, show it graphically, and calculate summary statistics [38]. This versatile easy-to-use tool uses the Monte-Carlo technique for calculating uncertainty and sensitivity and predict the entire range of the probable results for a specific situation [37]. Fig. 3 illustrates the CB ribbon in Microsoft Excel software.

Crystal Ball® (CB) ribbon is divided into five status bar that each of which relates to a step in the analysis or the modeling process; Define, Run, Analyze, Tools, and Help section. Define section contain all the elements which are needed to define the model and is applied for definition of variables; the middle section called Run contains all the items which are required to run and reset the simulation. The Analyze section contains the needed elements to report and analyze the result of the simulation. The Tools section contain additional tools which are applied to set up the run and analyze simulation as well as performance optimization and time series forecasting, and finally, the Help section includes many resources, for example, guides and documentation [38].

**Fig. 4:**
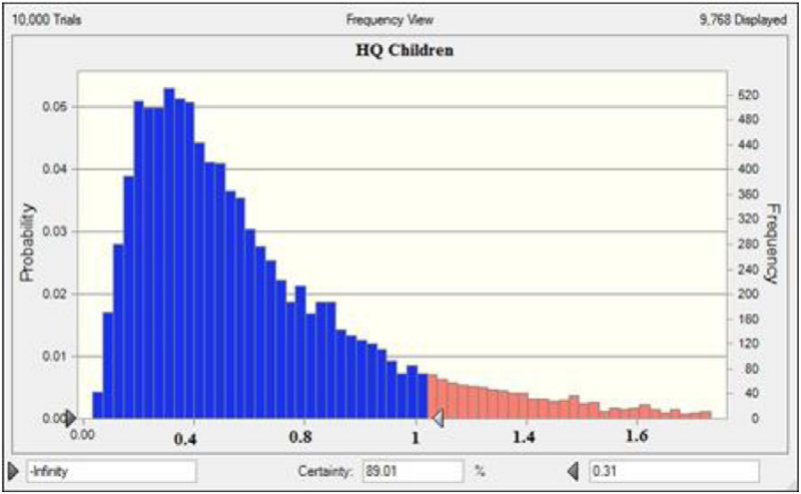
HQ values for children groups.

**Fig. 5:**
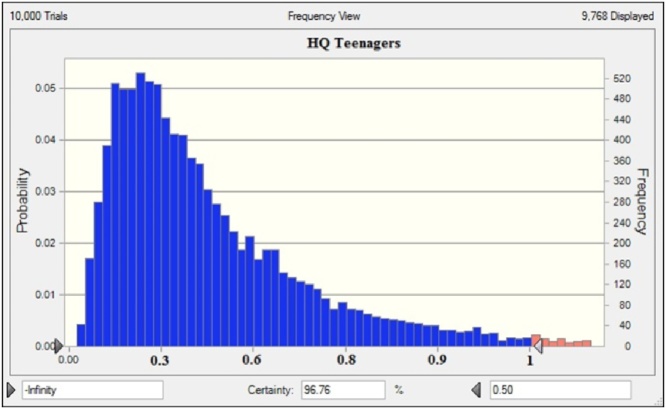
HQ values for teenager groups.

In the present research, Oracle Crystal Ball® software (Version 11.1.34190) was applied to simulation data and to estimate distribution parameters [17]. Fig. 4, Fig. 5 show the sensitivity analysis by using the Monte Carlo simulation technique; also, the HQ values have been indicated in Table 2.

As discussed later, the health risk assessment was carried out in four groups, including infant children, teenager, and adult, to investigate the non-carcinogenic risk of nitrate. The effect of probability estimation indicated that HQ levels in the studied groups increase in the order of children > teenager > adult > Infants. The results of the calculation of the point and probable hazard estimation are presented in Table 3.

**Table 3:** Deterministic and probabilistic approaches to determine HQ.

Parameter	Infant	Children	Teenager	Adult
*Deterministic approach*				
Mean	0.017	0.0165	0.0145	0.011
SD	0.006	0.007	0.005	0.004
P90	0.33	0.312	0.313	0.299

*Probabilistic approach*				
Mean	0.0171	0.0165	0.0144	0.010
SD	0.005	0.0068	0.0056	0.0039
P90	0.331	0.311	0.312	0.3

Besides, HQ levels of children and teenager groups found to be higher than one (1.7354 and 1.2250, respectively), indicating that they were the most sensitive groups in the studied exposed population. The simulation results with 90% confidence show that the HQ levels in the infant groups, children, teenagers, and adults are less than 0.331, 0.311, 0.312, and 0.3, respectively. So, according to results, long-term exposure to nitrate through drinking water consumption does not increase the likelihood of non-carcinogenic risk and the adverse effects of water consumption and exposure to nitrate, and the exposure to nitrate in the exposed population is safe during the present study period.

## Conclusion

Accurate and genuine information about drinking water pollutants is so vital in order to the promotion of social health programs. Since drinking water is one of the routes of human exposure to several elements, the consumption of water contaminated with nitrate could pose a health risk to the consumers. From this perspective, in the present study, 66 samples of groundwater resources of Iranshahr area were studied for the health risk assessment of nitrate by application of Monte-Carlo simulation (MCS) technique. The result of the present study showed that long-term exposure to nitrate through drinking water consumption does not increase the likelihood of non-carcinogenic risk. In the present research, children and teenager were more at risk by the consumption of polluted drinking water. Finally, it is recommended that groundwater resources be monitored and controlled more precisely to prevent the adverse health effect for consumers.

## Conflict of interests

The authors declare that there is no conflict of interest.
